# The Baltimore Academy of Medicine

**Published:** 1886-07

**Authors:** 


### THE BALTIMORE ACADEMY OF MEDICINE.

Officially Reported for THE ATLANTA MEDICAL AND SURGICAL JOURNAL.

STATED MEETING, HELD MAY 18, 1886.

### NIDINIZED PLATINA NEEDLES.

Dr. J. J. Chisolm exhibited a series of needles of various sizes, the invention of Dr. Genese, of this city, a description of which is here given in a note for the inventor.

They are made with platinized gold head, hardened under hydraulic pressure. This needle can be made as dense as steel, but will be found to work better if the temper is below spring steel. It will pass through tough tissue easily and cannot be broken, while the advantage of bending, if required, may be found of great service in some cases. This needle will pass through cartilage or dense cicatricial tissue. If irridium is added in the construction of this needle, it will be so hard that steel can be cut with it. This needle can be made of any shape or size, and it is indestructible under the pressure of the forceps or the action of acids. The eye of a needle is the part hardest to clean; this needle can be cleaned with nitric acid, thus making it a somewhat antiseptic needle.

Dr. S. C. Chew reported an interesting case of

### OPIUM POISONING.

He was called in consultation to see the patient, a lady who had been suffering from a severe attack of erythema nodosum, for the relief of which she had been getting a quarter of a grain of sulphate of morphia each night for four nights. On the fourth night she became comatose; a weak, irregular pulse set in and a Cheyne-Stokes respiration appeared. The weak, irregular pulse the doctor considered as contra-indicating poisoning by opium. She was at the time supposed to be beyond relief. Hypodermic injections of brandy and tr. of belladonna were, however, ordered, but gave no relief. At about five o'clock on the morning following, she was apparently past all aid, but as a last resort the attending physician gave her a hypodermic injection of tr. of belladonna alone of about twenty-five minims. To his surprise she began to rally, and by 9 o'clock of the same morning she was out of danger.

### MORPHIA TAKEN FOR QUININE—MERCURIAL INUNCTIONS FOR LICE.

Dr. F. T. Miles related a case of a man who was poisoned by taking morphia for quinine, and when seen was apparently so near dead that therapeutic aid was considered useless. The patient recovered without any treatment whatever. The doctor has never seen a patient die during the Cheyne-Stokes respiration.

Dr. Miles referred to a case of a young man, supposed to be syphilitic, who had become paralyzed. The man was completely paralyzed; there was dribbling of urine, bed sores and semi-unconsciousness. He was in a most disgusting condition, being filthy and in the hairy portions of his body infested with lice. Mercury was ordered internally for the syphilis and mercurial inunctions for the lice. The man made a rapid and complete recovery, which the doctor attributes to the rapidity with which his system was brought under the influence of the mercury—not so rapidly when given by the mouth as when rubbed into the surface; the object thus obtained was not intentional, as the inunctions were ordered as stated for the lice, but the result certainly suggests a valuable means of getting the rapid action of mercury.

Dr. J. J. Chisolm related a case of

OTORRHEA

that he had been called in consultation to see. The patient was in a very precarious condition. He was in a semi-comatose condition and was at times unconscious. The mastoid region was painful, but not swollen. Pus oozed from the ear. There was aphasia, but no paralysis. He decided to open the mastoid cells, but upon a second consideration, as the patient was apparently moribund, concluded to try the effects of a large blister to the nape of the neck, extending well down over the shoulders. The effect was pronounced; recovery was complete and the patient is now in good health.

